# Determining the potential scalability of transport interventions for improving maternal, child, and newborn health in Pakistan

**DOI:** 10.1186/s12961-015-0044-5

**Published:** 2015-11-25

**Authors:** Naeem uddin Mian, Mariam Zahid Malik, Sarosh Iqbal, Muhammad Adeel Alvi, Zahid Memon, Muhammad Ashraf Chaudhry, Ashraf Majrooh, Shehzad Hussain Awan

**Affiliations:** Contech International, 2-G Model Town, Lahore, 54700 Punjab Pakistan; Research & Advocacy Fund, Islamabad, Pakistan

## Abstract

**Background:**

Pakistan is far behind in achieving the Millennium Development Goals regarding the reduction of child and maternal mortality. Amongst other factors, transport barriers make the requisite obstetric care inaccessible for women during pregnancy and at birth, when complications may become life threatening for mother and child. The significance of efficient transport in maternal and neonatal health calls for identifying which currently implemented transport interventions have potential for scalability.

**Methods:**

A qualitative appraisal of data and information about selected transport interventions generated primarily by beneficiaries, coordinators, and heads of organizations working with maternal, child, and newborn health programs was conducted against the CORRECT criteria of Credibility, Observability, Relevance, Relative Advantage, Easy-Transferability, Compatibility and Testability. Qualitative comparative analysis (QCA) techniques were used to analyse seven interventions against operational indicators. Logical inference was drawn to assess the implications of each intervention. QCA was used to determine simplifying and complicating factors to measure potential for scaling up of the selected transport intervention.

**Results:**

Despite challenges like deficient in-journey care and need for greater community involvement, community-based ambulance services were managed with the support of the community and had a relatively simple model, and therefore had high scalability potential. Other interventions, including facility-based services, public-sector emergency services, and transport voucher schemes, had limitations of governance, long-term sustainability, large capital expenditures, and need for management agencies that adversely affected their scalability potential.

**Conclusion:**

To reduce maternal and child morbidity and mortality and increase accessibility of health facilities, it is important to build effective referral linkages through efficient transport systems. Effective linkages between community-based models, facility-based models, and public sector emergency services should be established to provide comprehensive coverage. Voucher scheme integrated with community-based services may bring improvements in service utilization.

## Background

Pakistan is the third largest contributor to the global toll of maternal mortality [1]. Despite the substantial progress made in reducing maternal and infant mortality, the country faces difficulties in reaching Millennium Development Goals 4 and 5 regarding the reduction of maternal mortality by 75% and under-five mortality by 66% between 1990 and 2015. Challenges include limited geographical access to healthcare facilities, poor physical infrastructure, lack of adequately trained and skilled staff, paucity of medical equipment and supplies, poor telecommunication mechanisms, and inadequate referral services [2]. The National Maternal, Newborn and Child Health (MNCH) Policy and Strategic Framework of Pakistan identified the absence of ambulance and telecommunications systems, difficulties in arranging private transportation, distance to nearest health facility, terrain, lack of information about location of the nearest secondary care facility, and cost considerations, as major barriers in causing delays in transportation [3].

Complications during pregnancy and birth may rapidly become life threatening, so timely access to emergency services by efficient transport services plays an important role in the survival of women and their newborns. According to Rose et al. [4], accessibility of transport and greater proximity to healthcare facilities are factors linked to lower levels of maternal and child deaths. Approximately 80% of maternal deaths can be averted if women have access to essential maternity and basic healthcare services [5]. National household surveys conducted in Pakistan [6–8] illustrate the link between proximity to health facilities and higher rates of utilisation of key MNCH services. Distance and high costs of transport are also documented as key factors which determine women’s use of services [9, 10].

In Pakistan, various transport initiatives have been implemented to improve accessibility of healthcare facilities during emergencies, including the Edhi Ambulance service, Rescue 1122, Agha Khan Health Services (AKHS) Gilgit, and Community Emergency Ambulance Service (CEAS) Ramthaman. These interventions have varied geographical coverage – ranging from a single union council to all provinces and regions of the country. The majority of these interventions provide free transport. Where fees are applicable, social protection is provided for those who are unable to afford the services. Our objective is to review existing transport interventions and assess their potential for scalability to improve MNH outcomes in poor and marginalised communities of Pakistan.

## Methods

### Mapping and data collection

A list of transport interventions working across Pakistan was developed through a mapping exercise based upon (1) review of existing literature, including reports, publications, and webpages on transport interventions implemented in all regions of Pakistan, and (2) electronic and telephonic communications with government officials at provincial and district levels, including Executive District Officers of Health, Provincial Director Generals of Health Services, Provincial Secretaries at the Department of Health, Provincial Managers of MNCH Programs, and public health experts working in national and international NGOs, donors/agencies, and development partner organizations.

We attempted to obtain information on the description of interventions which included its goals, distinguishing technical, organisational, and process elements, analysis of need or demand for the service among target populations, analysis of changes required in the intervention to make it applicable in other parts of the country, comparative analysis of associated costs, and evaluation reports [11]. These characteristics were, however, deficient in the majority of documentation found. To address this issue, information about the interventions was supplemented by face-to-face meetings with key informants, including organisational heads, operational managers, and beneficiaries.

A total of 35 key informant interviews (KIIs) were conducted, including seven KIIs with provincial managers of the MNCH program, seven KIIs with heads or representatives of implementers of transport interventions, 14 with operational managers of transport interventions, and seven with drivers of transport vehicles. In addition, 14 focus group discussions (FGDs) were conducted with beneficiaries (mainly women of childbearing age) and their husbands to obtain information for our appraisal. Data was collected across north, south, and central regions of Pakistan. Qualitative guides were used for interviewing key informants to gain in-depth insights on objectives, scope of transport interventions (coverage and target population), implementation arrangements, operational and management costs, relevance to community need, operational features, service delivery, observability and effectiveness, monitoring mechanisms, scaling up, and transferability.

### Categorisation and selection

Based on the above information, we divided the interventions into four major categories: community-based services, facility-based services, public sector emergency services, and transport voucher schemes. The characteristics of the interventions were analysed according to the following parameters (Table 1): (1) concept – to identify the focus and objectives of the intervention; (2) geographical location – to select the intervention from all the three identified regions (North, Centre, and South) of the country; and (3) management categorization – in order to cater to the various management techniques employed for implementation of transport interventions. The following criteria were then used to select a short-list of interventions: (1) at least one intervention from each category; (2) geographical representation, with at least one from each region; (3) the most commonly utilized transport initiatives according to community respondents; (4) availability of relevant documentation describing the intervention; and (5) willingness of initiatives to be included in the study by verbal and written consent.

**Table 1:** Short-listed transport interventions for scalability assessment

Categories of services	Sr. #	Name of intervention	Concept	Geographical location	Management categorisation
Community-based services	1.	Ambulance initiative of Aga Khan Health Services (AKHS)	Transport services for all types of emergency and patient referral in hard-to-reach areas	North (Gilgit Baltistan)	NGO, free services
	2.	Community Emergency Ambulance Services	Transport services to all types of emergencies with a focus on maternal, newborn, and child healthcare (MNCH) and community awareness component	Centre (Punjab)	Community partnership, fee for services and social protection for the poor
	3.	JORDAN (Johi Organization for Rural Development and Natural Disaster)	Community-based patient transportation services in district of Dadu Sindh with a focus on MNCH	South (Sindh)	NGO, free services
	4.	Community Balochistan Ambulance Services	Provision of services for all types of emergency and patient referral in rural areas of district Lasbela	South (Balochistan)	NGO, free services
	5.	Rural Emergency Ambulance Service Initiatives (RESAI)	Provision of emergency transport services focusing on MNCH in rural areas of five selected PAIMAN districts of Pakistan, i.e. Vehari, Multan, Dadu & Jafferabad, DG Khan	All regions of Pakistan	Community partnership, fee for services and social protection for the poor
	6.	Edhi Ambulance Services	Transport services for all types of emergency services in all districts/regions of Pakistan	All across Pakistan	NGO, free services
	7.	Chipa 1020	Transport services for all types of emergencies in district Karachi	Sindh (South)	NGO, free services
	8.	Ambulance and Coffin Carrier Services	Transport services for all types of emergencies in district Karachi and Lahore	Sindh (South)	NGO, free services
				Center (Punjab)	
	9.	Ambulance Services	Provision of transport services for all types of emergencies in urban and periurban areas of district Khuzdar	South (Balochistan)	NGO, free services
	10.	Ambulance Services	Provision of transport services for all types of emergencies in 12 rural districts of Balochistan	South (Balochistan)	NGO, free services
	11.	Ambulance Services	Provision of transport services for all types of emergencies in urban and periurban areas of 14 districts of Balochistan	South (Balochistan)	NGO, free services
	12.	Patient Ambulance Services	Transport services for all types of emergencies in ICT and Rawalpindi district (Punjab)	Center (ICT, Punjab)	NGO, free services
	13.	Ambulance Services	Transport services with focus on MNCH services in urban and rural areas of district Larkana and Nawab Shah, Sindh	South (Sindh)	NGO, free services
	14.	Community ambulance interventions	Transport services for all types of emergencies for rural areas of district Khanewal, Punjab	South (Balochistan)	District Govt. free services
	15.	Community based interventions	Transport services focusing on MNCH for district Khuzdar, Balochistan	South (Balochistan)	NGO/Public-private partnership, free services
Public sector emergency services	16.	Rescue 1122 Punjab	Transport services for all types of emergencies in urban and periurban area in all 36 districts of Punjab	Centre (Punjab)	Public sector, free services
	17.	Rescue 1122 KP	Transport services for all types of emergencies in urban and periurban area in district Peshawar and Mardan	North (KP)	Public sector, free services
Facility-based services	18.	CHARM Initiative	Based at selected BHUs, providing transport services to catchment population with a focus on MNCH	Centre (Punjab)	Public sector, free services
Transport voucher schemes	19.	Family Health Insurance Initiative (Sehat Sahulat Scheme-SSS)	Provision of emergency transport services to beneficiaries of health insurance initiative	South (Balochistan)	NGO, fee for services (paid by insurance agency)
	20.	Voucher Scheme (NPPI)	Provision of vouchers for utilisation of transport services for maternal healthcare	South (Sindh)	NGO, free services for poor beneficiaries
	21.	Health voucher	Provision of vouchers for utilisation of transport services focusing on MNCH for rural beneficiaries in district DG Khan	Center (Punjab)	NGO, free services for poor beneficiaries
	22.	Health voucher	Provision of vouchers for utilisation of transport services focusing on MNCH for rural beneficiaries in district Jhang	Center (Punjab)	NGO, free services for poor beneficiaries

### Assessment of scalability

Qualitative appraisal was conducted using a checklist based upon CORRECT criteria [12], exploring the Credibility, Observability, Relevance, Relative Advantage, Easy-Transferability, Compatibility, and Testability, which are recognized critical elements for assessing scalability potential. The presence or absence of key elements of CORRECT criteria in each selected transport intervention was determined in the following manner: (1) credibility was assessed on grounds of the intervention tested in similar settings coupled with availability of evidence and external evaluation of the intervention by a third party; (2) observability was seen in terms of results being visible to the general population, and a clear association between results and objectives of the intervention; (3) relevance was determined based on demand, i.e. whether need is sharply felt by the population using the services as well as perceptions of government officials; (4) relative advantage was assessed on the basis of whether current solutions in the area were sufficient, as well as any availability of evidence on the cost effectiveness of interventions, as compared with other solutions; (5) easy transferability – an intervention was considered easy to transfer if it was only a slight deviation from current practices of government, had a simple model with low technical sophistication, was able to use current infrastructure and facilities, and generated revenue. Technical sophistication was determined on basis of the specifications of vehicle used, availability of staff/equipment in the ambulance, and training requirements for paramedical staff and revenue generation; (6) compatibility was considered if it was in line with established norms and beliefs of its beneficiaries, i.e. the beneficiaries considered it to be acceptable and culturally appropriate; and (7) testability was explored on whether it could be tested by government or development partners on a limited scale.

Collected data was analysed by adapting the qualitative comparative analysis (QCA) technique [13], which involves analysing interventions against combinations of operational indicators based upon CORRECT criteria. Logical inference was drawn to assess the implications of each intervention. Cross-case trends were explored by comparing interventions using QCA, keeping in view the diversity and heterogeneity of each intervention.

To analyse the scalability potential, QCA was used as a crude test. Presence of a CORRECT element that simplifies or complicates scaling up was scored as 1, whereas its absence was assumed to be 0. An intervention’s scalability potential was roughly measured by accumulating the scores for simplifying factors and subtracting scores earned for complicating factors. The higher the score of an intervention, the more potential for scaling up. This was further strengthened on the basis of findings of KIIs and FGDs, which were used by the authors to re-examine and revisit data and provide explanation and reaffirmation of the given scores against each criterion.

### Ethical considerations

Informed and verbal consent were sought for KIIs and FGDs. The confidentiality and anonymity of participants was maintained by coding each participant with a unique ID. The study was reviewed and approved by the National Bioethics Committee of the Research & Advocacy Fund.

## Results

The 22 interventions initially found were placed into four major categories: community-based services, facility-based services, public sector emergency services, and transport voucher schemes (Table 1). Of these, seven interventions were selected, as detailed below.

### Community-based services

The transport schemes included in this category were those where a small van served as an ambulance stationed in the community. The services were operated with the support of the local community under the supervision of the associated program. Three were selected based on our predefined criteria: (1) The Community ambulance service in Gilgit, Baltistan, a non-government initiative implemented in Valley Hunza Nagar to improve access of the population to health services for all emergency cases. The van-type ambulance operated in a geographically difficult terrain where other available modes of transport were limited and expensive. (2) The CEAS, a non-government initiative aiming to cater for the emergency health transport needs of the community, particularly to prevent delays in reaching health facilities in emergency obstetric cases in eight villages of Union Council Matta, District Kasur Punjab. Bolan-van type CEAS provided 24 hour, 7 day services to the community to transfer patients from home to a hospital or health facility. (3) The Johi Organization for Rural Development and Natural Disaster (JORDAN) Community Ambulance Service is a non-government initiative covering 30 Union Councils of District Dadu, Sindh, to manage obstetric and neonatal complications by improving the readiness of health facilities through a referral system and overcoming delays in reaching a health facility.

### Facility-based services

Facility-based services included interventions in which the ambulance is based at a public sector health facility. One initiative was identified, the so-called ‘CHARM’ Ambulance Service, a government initiative under the Chief Minister’s Health Initiative for Attainment & Realization of Millennium Development Goals (CHARM). It is implemented in 89 selected Basic Health Units (BHUs) of seven districts of Punjab, aiming to provide timely access to quality transport services to manage MNCH-related emergency cases from the community to BHUs and from BHUs to higher referral health facilities. The initiative provides modified vans as ambulances, located at BHUs.

### Public-sector emergency services

Government-established public sector emergency services were included in this category as part of the first responders in case of any emergency situation. The services focused on all types of emergencies and had a well-known and good response rate. One initiative was selected, namely the Rescue Services 1122 Chakwal, a Government-led initiative to provide emergency transport services to all emergency cases requiring transportation, especially in case of road traffic accidents and also for MNCH services. A well-equipped van was provided along with trained staff within a centralized base station. Its telecommunication and management system allows maintenance of an average response time of 7 min to reach into the community.

### Transport voucher schemes

Transport voucher schemes were identified as interventions where vouchers were provided to the poor and marginalised members of the population for services, which included re-imbursement for transport costs. Two such interventions were selected: (1) the transport voucher scheme under the Norwegian-Pakistani Partnership Initiative (NPPI), a non-government initiative in Badin and Shikarpur districts providing incentives to pregnant women living below the poverty line for antenatal, postnatal, and institutional delivery care utilization. Transportation costs were provided to the beneficiaries to access the network of private sector providers and some public sector facilities for referral care; and (2) the Sehat Sahulat Scheme (SSS), a non-government initiative intended to provide families living below the poverty line with access to free-of-cost health services. This voucher involved transportation of pregnant women, trauma cases, and other emergency cases to health facilities.

A scalability assessment was conducted to gauge the potential of the seven selected transport interventions. Table 2 highlights the appraisal findings of each transport intervention in relation to CORRECT criteria and the simplifying and complicating factors. Rescue 1122 Chakwal (a public sector model) secured the highest score of 6. Two community-based interventions (CEAS, JORDAN) and a health facility intervention (CHARM) received a score of 4. The score for AKHS was 2 and negative scores were received by SSS and NPPI.

**Table 2:** Scalability assessment of selected seven transport interventions against CORRECT criteria

Criteria	Simplifying factor	Interventions							Complicating factor	Interventions						
		Public sector	Community based			Facility based	Transport vouchers			Public sector	Community based			Facility based	Transport vouchers	
		RS	CEAS	JORDAN	AKHS	CHARM	SSS	NPPI		RS	CEAS	JORDAN	AKHS	CHARM	SSS	NPPI
Credibility	Model based on sound evidence	1	0	1	0	0	0	1	Little or no solid evidence	0	1	0	1	1	1	0
	Model evaluated by third party	1	0	0	0	0	0	0	Not evaluated by independent sources	0	1	1	1	1	1	1
Observability of results	Results visible to general public	1	1	1	1	1	1	1	Not very visible; not easily communicated to public	0	0	0	0	0	0	0
	Clearly associated with objectives	1	1	1	1	1	0	0	Not clearly associated with intervention	0	0	0	0	0	1	1
Relevance	Addresses demand sharply felt by population	1	1	1	1	1	1	1	Addresses a need not sharply felt by population	0	0	0	0	0	0	0
	Addresses demand sharply felt by government	1	1	0	0	1	0	0	Addresses a need not sharply felt by government	0	0	1	1	0	1	1
Relative advantage	Current solutions considered sufficient	1	1	1	1	1	1	1	Current solutions are considered adequate	0	0	0	0	0	0	0
	Superior cost-effectiveness as compared to current solutions	0	0	0	0	0	0	0	Little or no objective evidence of superiority to current solutions	1	1	1	1	1	1	1
Easy to transfer	Small difference from current practices for government	1	0	0	0	0	0	0	Large difference from current practices for government	0	1	1	1	1	1	1
	Simple model having low technical sophistication	0	1	1	1	1	0	0	Complex model having high technical sophistication	1	0	0	0	0	0	0
	Able to use current infrastructure and facilities	0	0	0	0	1	0	0	Requires new infrastructure and facilities	1	1	1	1	0	1	1
	Revenue generation	0	1	1	1	0	0	0	No revenue generation	1	0	0	0	1	1	1
Compatible	In line with beneficiaries’ established norms and values	1	1	1	1	1	1	1	Not in line with beneficiaries’ established norms and values	0	0	0	0	0	0	0
Testability	Able to be tested by government on a limited scale	1	1	1	1	1	1	1	Unable to be tested without complete adoption	0	0	0	0	0	0	0
SCORE		10	9	9	8	9	5	6		4	5	5	6	5	8	7
RESULTS (simplifying-factor score – complicating-factor score)		6	4	4	2	4	−3	−1								

RS, Rescue Services 1122 in Chakwal, Punjab; CEAS, Community Emergency Ambulance Services Punjab; CHARM, CHARM initiative: BHU-based ambulance services in Punjab; JORDAN, Community Ambulance services in Sindh; AKHS, Community Ambulance Services under Agha Khan Health Services in Gilgit Baltistan; SSS, Sehat Sahulat Scheme Vouchers for transportation in Balochistan; NPPI, Voucher scheme for transportation under NPPI in Sindh

Rescue 1122 Chakwal, JORDAN, and NPPI were found more credible as the design of the interventions was based upon proven models. The respondent (KII-18) indicated that the Rescue 1122 model was initially launched in Lahore district in 2004 as a pilot project. The project was perceived as a success and led to its expansion in all 36 districts of Punjab. He further explained that this project had also been evaluated by a third party (Punjab Economic Research Institute), adding to evidence of its credibility.

The KIIs and FGDs provided further information regarding observability of results. Visibility of transport interventions to the general population was highlighted, coupled with perceptions of increased utilization of services by mothers. The majority of mothers who used the services mentioned that they had received these ambulance services free-of-cost with easy access. A mother (FGD-M22) who availed CEAS ambulance service in Kasur district stated:

“*Before the launch of CEAS, people, especially pregnant females, had no choice except to use private transport, where affordability remained a concern. In my view, CEAS is an affordable and easily accessible service*.”

Regarding the relevance of transport interventions in addressing the needs of the population as well as the government, CEAS, Rescue 1122, and CHARM were found in KIIs and FGDs to have characteristics of relevance. For example, the ambulance service established under the CHARM project addressed the issues of timely availability of transport in rural areas as stated by respondent KII-11:

“*In 2011, 3,160 MNCH patients were referred from basic EmONC to comprehensive EmONC health facilities using this service while in 2012, from January to September, the number of referred patients was 3,228. Out of 23,155 deliveries occurring at health facilities, 16,000 were brought from their homes to the health facilities on this ambulance service.*”

Considering the factors of relative advantage, all seven transport interventions were providing adequate solutions to cater for the needs of the community by making services available, accessible, and affordable as mentioned by KII-18:

“*Rescue 1122 is the only specialised transport emergency service compared to other transport solutions in Punjab, having an average response time of 7 min.*”

However, none of the transport interventions were able to provide evidence for cost-effectiveness. It was perceived that such interventions required huge funds for operationalization. One interviewee (KII-14) highlighted that managing the SSS ambulance services in Lasbela required more professional staff, community mobilizers, and better equipment and finances to make the service more effective. He further stated:

“*In order to cater to 1500 families in Lasbela, huge investment into operationalization was made.*”

In terms of transferability, five transport interventions (CEAS, RS, CHARM, JORDAN, and AKHS) were able to meet the criteria of ‘easy to transfer’. Conversely, Rescue 1122 was a novel concept for emergency services and deviated from traditional Government practices, while also having complex technical specifications, including vehicles with specialised equipment and tracking systems, a control room, paramedics in each ambulance, and the need for new infrastructure and equipment for its control room and call centre, which were hindrances in transferability. Furthermore, the cost of services had to be borne by the government, placing an extra financial burden on the exchequer. CEAS, JORDAN, and AKHS, on the other hand, had relatively simple organisational structures and low technical sophistication, which could be scaled up effectively, especially if coupled with revenue generation (as done by CEAS and JORDAN), which could reportedly help in the sustainability of the intervention. The CHARM ambulance service was the only initiative which was described as able to use existing infrastructure and facilities, i.e. the BHU and linkages with the community through the lady health workers, making it more transferable in nature.

All the seven transport interventions were found compatible with cultural norms and values of beneficiaries residing in North, Central, and South regions of Pakistan, having linkages within communities and with healthcare providers. FGD respondents in Kasur district reported that they were advised by traditional birth attendants and lady health workers to call the CEAS in case of an emergency. This positive feedback by community members indicated their trust on CEAS was in line with the established norms and values of the community. Emphasis was also given on respect to women and communication with patients in the native language while transporting them to the health facilities. A driver (KII-30) said,

“*I talk softly with patients in Punjabi language so that they may communicate with me easily. Upon shifting females, particularly pregnant patients, in ambulance, I take care of their ‘pardah’ and ensure they are accompanied by their family members.*”

KII participants, including heads of organizations, operational managers, and officers in-charge, affirmed the testability of the seven interventions stating that they have the potential to be tested out by the Government or other development partners on a limited scale within the same geographical settings.

## Discussion

Scaling up refers to increasing the effects of innovations in health services that have been successfully tested in pilot or experimental interventions, with the aim of benefitting more people, as well as for the development of a program on a long-term basis [14]. In order to consider any pilot intervention for scaling up, its scalability potential needs to be assessed. Scalability denotes the potential of the intervention to be implemented on a large scale, which can be assessed using specific criteria. In this study, the scalability of seven selected transport interventions was assessed against CORRECT criteria, using the QCA technique. However, the authors acknowledge facing limitations in comparing interventions which were not similar and which varied in design, concept, and operationalization. A dearth of uniform and standard documentation for each intervention was also experienced. The research explores the potential of scalability in four main categories of interventions, highlighting their strengths and weaknesses.

The provision of ambulance services within health facilities is an indicator of clinical quality and prompt response aimed at saving lives [15]. Facility-based services have the added advantage of availability of infrastructure throughout the country and the ease of establishing linkages across health facilities, which could be considered positives of this model. However, there are certain factors which affect the overall potential for up-scaling of these services. For example, this model requires functional health facilities round the clock, so as to serve as base stations for ambulances. Without fulfilling this pre-requisite, ambulance services may not be fully operational. In view of the poor infrastructure of public sector health facilities in Pakistan, particularly at primary level (BHUs), where these ambulances were to be based, the scenario is not promising. A large number of health facilities, especially BHUs, are non-functional and will pose serious limitations to this model [16]. Furthermore, in the past, ambulances were provided at Rural Health Centres for referral and transportation; however, this initiative has remained limited due to the inadequate managerial and leadership capacity of the public sector [17]. These issues, coupled with financial burden on the exchequer for the sustainability of this model, adversely affect the scalability potential of the model.

Transport voucher schemes addressed affordability issues, as well as improving access to health services of disadvantaged members of society, ensuring the availability of free transportation during emergency medical need for poor and vulnerable families. Many countries have tested out this model, e.g. Kenya, Uganda, Bangladesh, among others [18, 19]. However, in the context of this research, there are some key challenges related to the selected voucher schemes, which reduced the potential for up-scaling of this model. The voucher scheme provided the option of re-imbursement of transport costs; however, it could not ensure the availability of transport. Moreover, it cannot operate as a stand-alone intervention focused only on transport. It would be possible to provide a complete package of services, which should also include the option of transport. However, such a scheme would require extensive resources and technical expertise for voucher management. In addition to this, the possibility of collusion and fraud in voucher management also negatively affects the potential of up-scaling for this model [20].

The government established public sector services as part of the first responders in case of any emergency situation, focusing on all types of emergencies, including road traffic accidents. The services have a very good response rate and well-equipped vehicles with trained staff in every unit. We found that Rescue 1122 Chakwal has more potential for scalability according to the CORRECT criteria, coupled with best on-journey care as well as demonstrated quick response times. However, high technical specifications, as well as the need for new infrastructure and equipment, the financial burden on the exchequer, and questionable long term sustainability are of high concern and can become a liability in terms of scaling up of services. If these requirements were not to be fulfilled, the quality of services would suffer, resulting in a decrease in quality of services and de-meriting of the public sector emergency services.

The model of community-based services is a strong and resilient model in communities where the relationship between the ambulance services and the community is socially constructed and where advanced technology, rules, systems, procedures, and policies cannot be sustained [21]. This design has been successfully used in many low-income countries like Gambia, Malawi, Ghana, and Tanzania [22]. Furthermore, various examples of community-based services demonstrated that such designs, with low technical specifications and free-of-cost services, encourage sustained community support due to a feeling of ownership [23]. The relatively simple design, free-of-cost services, community ownership and participation, and financial sustainability through revenue generation, contributions, and donations, give an extra edge to this model. Deficient in-journey care and ensuring community involvement on a long-term basis have been assessed as challenges being faced by these models. Our findings and the available literature suggest that community-based services seem to have the highest scalability potential. Improving the in-journey care component can further strengthen this model. Further, integrating a voucher scheme for healthcare services at a facility with a community-based service could help in improving utilisation of the services.

## Conclusions and recommendations

A one-fit-for-all solution may not be the answer to overcome multiple MNCH-related transport barriers. Interventions with the highest potential for scalability may not be feasible to cater for all variations in transport needs, so integration of multi-faceted interventions may have to be considered as a solution. Findings of this study suggest that the community-based services model has good features for coping with MNCH-related emergencies. It is suggested that this model could be used to provide transport services in uncovered areas in Pakistan, and it should be strengthened with the help of other transport models. In order to strengthen this model, effective linkages between community-based models, facility-based initiatives, and public sector emergency services should be established to provide comprehensive coverage. Cheaper transport alternatives should be considered in areas where no affordable transport is currently available.

A transport system should thus be planned as part of a long-term integrated approach to make the health system more effective in reducing maternal and child morbidity and mortality.

## Abbreviations

AKHS: Agha Khan Health Services
BHU: Basic Health Unit
CEAS: Community Emergency Ambulance Service
CHARM: Chief Minister Health Initiative for Attainment and Realization of Millennium Development Goals
CORRECT: Credibility, Observability, Relevance, Relative Advantage, Easy-Transferability, Compatibility and Testability
FGD: Focus Group Discussions
JORDAN: Johi Organization for Rural Development and Natural Disaster
KIIs: Key Informant Interviews
MNCH: Maternal, Newborn and Child Health
NPPI: Norwegian-Pakistani Partnership Initiative
QCA: Qualitative Comparative Analysis
SSS: Sehat Sahulat Scheme
